# The clinico-pathological profile of non-Hodgkin’s lymphoma in Aseer region of Saudi Arabia

**DOI:** 10.1186/s13104-019-4447-1

**Published:** 2019-07-15

**Authors:** Nawaf Alyahya, Balkur Adiga, Ali Alwadei, Ghanem Alshahrani, Fahad Alyahya

**Affiliations:** 1 Department of Internal Medicine, King Abdulaziz Medical City, Riyadh, Saudi Arabia; 2 Department of Pathology, King Khalid University, Abha, Saudi Arabia; 3 College of Medicine, Imam Abdulrahman Bin Faisal University, Dammam, Saudi Arabia; 4 Pathology Lab, King Abdullah Hospital, Bisha, Saudi Arabia; 5 College of Medicine, Bisha University, Bisha, Saudi Arabia

**Keywords:** Non-Hodgkin lymphoma, NHL, Clinico-pathologic, Saudi Arabia

## Abstract

**Objective:**

Non-Hodgkin lymphomas (NHL) are a group of neoplastic lymphoproliferative disorders, in which, its clinical spectrum, primary extra nodal variety, histopathology and Immunohistochemistry, remain lacking in Saudi Arabia. We aimed to assess the clinicopathologic patterns of NHL and the utility and diagnostic role of IHC immunophenotyping.

**Results:**

Patients > 60 years of age had the highest incidence of NHL; male: female ratio was 1.27:1. The incidence of NHL has shown a steady increase in the Aseer region from 2011 to 2014. Twenty-Five percent of our patients presented with advanced disease (Stage IV). A total of 52% of patients presented with constitutional symptoms, while 43% showed generalized lymphadenopathy. Nearly half of our patients (49%) had primary NHL of extra nodal variety, where the stomach was the most commonly involved organ (13 cases). Diffuse large B cell lymphoma was the most common subtype of NHL in our population (59%). Most patients (82%) were positive for CD20 surface marker, while 60% were positive for CD45.

## Introduction

Non-Hodgkin lymphomas are a diverse group of neoplastic disorders, the incidence of which has shown a significant increase in rates over the year, especially in Saudi Arabia. In 2008, NHL was known as one of the most prevalent types of cancer in Saudi Arabia, with a second in rank in cancer incidence among male population (male to female ratio = 122:100) [1]. During the period from January 2009 to December 2010, the Saudi Cancer Registry recorded a total of 26,960 cases of cancer, of which a total of 1412 cases of NHL were identified, accounting for 7.2% of all cancer incidences and ranking the third [2]. More recently, the International Agency for Research on Cancer estimated an age-standardized incidence rate (ASIR) of NHL of 6.5 per 100,000 male patients with an age-standardized mortality rate (ASMR) of 4.3 per 100,000 male patients [3].

Extra nodal NHL has been reported to occur in 15 to 25% of all NHL patients in the United States (USA) and 30 to 42% in different parts of Europe [4–8]. Every single organ can be a targeted site for NHL of extra nodal variety. Data regards the incidence of primary extra nodal NHL, especially in the Saudi Arabia, remain limited.

Furthermore, diffuse large B cell lymphoma (DLBCL) has been shown to be the most frequently identified subtype of NHL [9]. Even though DLBCL is defined by the World Health Organization as a single disease entity, the variety of clinical encounter as well as genetic characteristics suggest that these neoplastic proliferations denote a dissimilar group of neoplasms [10].

Immunohistochemistry (IHC) has set the way for possible understanding of the pathogenesis of NHL as well as aiding in identifying the immunophenotype of most NHL cases [11]. Recently, IHC has become an important step in the investigation of diagnostic pathologic studies of NHL. It is used for classifying lymphatic neoplasms into B cell and T-cell phenotypes as well as for differential diagnosis with other malignant proliferations. CD20 is a cell surface marker expressed precisely on a majority of human B-cells [12]. Moreover, it has also been reported to be expressed on more than 90% of B-cell lymphomas; thus, it has become a good molecular goal for monoclonal antibody therapy [13, 14].

Due to the limited data of the clinical presentation and investigation of NHL in Saudi Arabia, a thorough insight into the clinical variety of NHL is essential for proper diagnosis and optimum treatment.

## Main text

### Methods

#### Study design and site

This is a chart review analysis of the medical records of 100 cases diagnosed with NHL during the period from January 2011 to December 2016 at the histopathology department, Aseer Central Hospital (ACH). ACH is the referral tertiary care hospital catering the Aseer province with a population of 3.8 million individuals.

#### Study population

All patients who were diagnosed with NHL in the period from 2011 to 2016 in our setting were included. Patients who were ineligible according to the above criteria were excluded.

#### Data gathering

Sociodemographic data in terms of age, sex, site of biopsy was retrieved from the medical records of NHL patients. Laboratory records as regards hemoglobin (Hb) level, White Blood Cell (WBC) count, and platelet count were also reviewed. Mild anemia was defined as Hb level from 9 to 12 g/dL, whereas moderate anemia was defined when Hb level ranged from 6 to 8 g/dL. Leucopenia was considered when WBC count was below 4000 cells/mm^3^, while leukocytosis was defined when WBC count was above 11,000 cells/mm^3^. Thrombocytopenia was defined as a platelet count below 150,000 cells/mm^3^.

Immunohistochemistry (IHC) data was reviewed to determine the phenotype of NHL cases into B-cell and T-cell lymphomas and their subtypes. The available markers that were examined included CD3, CD20, CD30, CD8, CD5, CD15, CD45, CD43, CD21, CD23, CYCLIN D1, BCL2, BCL6, CD38, CD10, CD99, CD79, CD110, CD68, PG, M1B1, Ki 67, Vimentin, EMA, and PAX5. Patients were administered chemotherapy regimens including CHOP (Cyclophosphamide, Adriamycin, Prednisolone, and Vincristine) only, or CHOP in conjunction with other chemotherapeutic regime, or chemotherapy other than CHOP. Other chemotherapeutic regimens included BACOP (Bleomycin, Adriamycin, Cyclophosphamide, Prednisolone, and Vincristine), CVP (Cyclophosphamide, Vincristine, and Prednisolone), ProMACE (Procatbazine, Methotrexate, Adriamycin, Cyclophosphamide, and Etoposide), and Cytoboin.

### Results

**Fig. 1:**
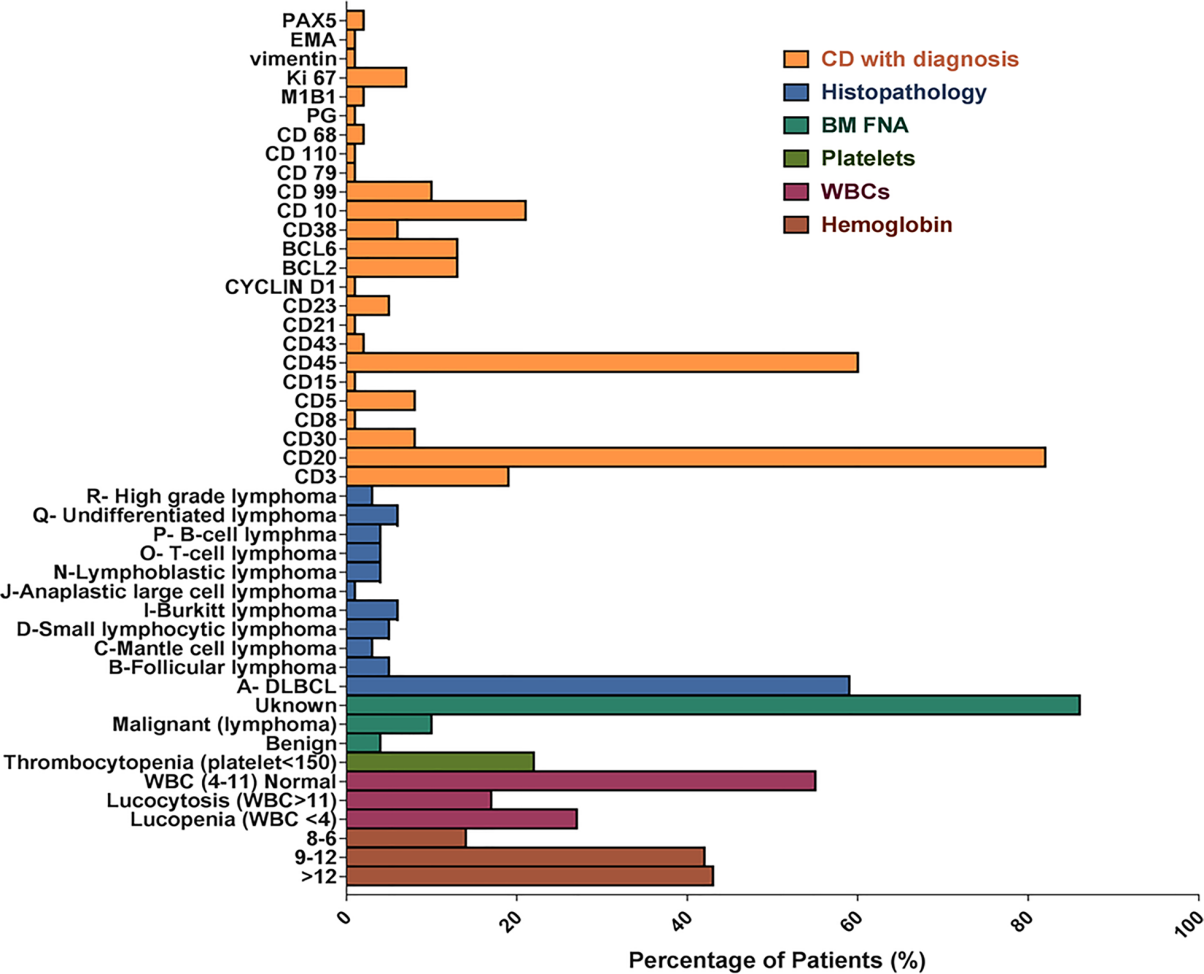
Histo-pathological and lab profile of included patients (N = 100)

The records of a total of 100 cases of NHL during the period from 2011 to 2016 were retrieved and analyzed (Fig. 1). Of which, NHL was predominant in males with a percentage of 56% as compared to females (44%). A steady increase in the number of cases of NHL from 2011 to 2014 (from 8 cases to 22 cases) while there was a reduction in 2015 (16 cases) which increased in 2016 to reach a total of 19 cases. Upon stratifying the incidence of NHL according to age groups, NHL occurred at all age groups, however, the age group (> 60 years) was the most affected group (35%) followed by the age group from 40 to 60 years (30%). Exposure to known carcinogens, family history of lymphoma and history of any genetic diseases were unknown in most of our cases 93%, 56% and 56% respectively (Table 1).

**Table 1:** Socio-demographic characteristics of included patients (stratified by year) (N = 100)

	Year														P-value ^¥^
	2011		2012		2013		2014		2015		2016		Total		
	N	%	N	%	N	%	N	%	N	%	N	%	N	%	
*Age*															
0–20 years	2	25	4	28.57	3	14.29	2	9.09	2	12.5	1	5.26	13	13	0.362
21–40 years	0	0	3	21.43	5	23.81	7	31.82	3	18.75	4	21.05	22	22	
41–60 years	2	25	4	28.57	7	33.33	4	18.18	8	50	5	26.32	30	30	
> 60 years	4	50	3	21.43	6	28.57	9	40.91	3	18.75	9	47.37	35	35	
*Sex*															
Female	3	37.50%	7	50.00%	11	52.38%	9	40.91%	6	37.50%	8	42.11%	44	44.00%	0.932
Male	5	62.50%	7	50.00%	10	47.62%	13	59.09%	10	62.50%	11	57.89%	56	56.00%	
*Nationality*															
Egyptian	0	0	1	7.14%	0	0	1	4.55%	1	6.25%	0	0	3	3.00%	< 0.001**
Indonesian	0	0	1	0	0	0	0	0	0	0	1	0	2	0	
Saudani	0	0.00%	0	0.00%	0	0.00%	1	4.55%	0	0.00%	0	0.00%	1	1.00%	
Saudi	7	1	11	1	19	1	20	1	13	1	17	1	87	1	
Syrian	0	0.00%	0	0.00%	0	0.00%	0	0.00%	1	6.25%	0	0.00%	1	1.00%	
Yemni	1	0	1	0	2	0	0	0	1	0	1	0	6	0	
*Occupation*															
Doctor	0	0.00%	0	0.00%	0	0.00%	0	0.00%	1	6.25%	0	0.00%	1	1.00%	0.005*
Driver	0	0.00%	0	0.00%	0	0.00%	0	0.00%	0	0.00%	1	5.26%	1	1.00%	
Housewife	0	0.00%	3	21.43%	7	33.33%	2	9.09%	3	18.75%	4	21.05%	19	19.00%	
Intermediate	0	0.00%	1	7.14%	0	0.00%	0	0.00%	0	0.00%	0	0.00%	1	1.00%	
Maid	0	0.00%	0	0.00%	0	0.00%	0	0.00%	0	0.00%	1	5.26%	1	1.00%	
Manual worker	0	0.00%	0	0.00%	0	0.00%	0	0.00%	1	6.25%	0	0.00%	1	1.00%	
Office job	0	0.00%	0	0.00%	0	0.00%	0	0.00%	0	0.00%	1	5.26%	1	1.00%	
Private job	0	0.00%	0	0.00%	0	0.00%	1	4.55%	0	0.00%	0	0.00%	1	1.00%	
Student	0	0.00%	1	7.14%	0	0.00%	0	0.00%	0	0.00%	1	5.26%	2	2.00%	
Retired	0	0.00%	0	0.00%	1	4.76%	0	0.00%	1	6.25%	0	0.00%	2	2.00%	
Other/not working	8	100.00%	9	64.29%	13	61.90%	19	86.36%	10	62.50%	11	57.89%	70	70.00%	
*Exposure to known carcinogens*															
Chronic liver disease + bilharsesis	0	0.00%	0	0.00%	1	4.76%	0	0.00%	0	0.00%	0	0.00%	1	1.00%	0.566
Smoking	0	0.00%	0	0.00%	1	4.76%	2	9.09%	0	0.00%	0	0.00%	3	3.00%	
Hepatitis c +ve	1	12.50%	0	0.00%	0	0.00%	1	4.55%	0	0.00%	0	0.00%	2	2.00%	
liver cirrhosis	0	0.00%	0	0.00%	0	0.00%	1	4.55%	0	0.00%	0	0.00%	1	1.00%	
Other/Unknown	7	87.50%	14	100.00%	19	90.48%	18	81.82%	16	100.00%	19	100.00%	93	93.00%	
*Family Hx of lymphoma*															
NO	8	100.00%	13	92.86%	0	0.00%	1	4.55%	3	18.75%	19	100.00%	44	44.00%	< 0.001**
Other/Unknown	0	0.00%	1	7.14%	21	100.00%	21	95.45%	13	81.25%	0	0.00%	56	56.00%	
*Family Hx of other cancer*															
HCC	0	0.00%	0	0.00%	0	0.00%	1	4.55%	0	0.00%	0	0.00%	1	1.00%	< 0.001**
liver carcinoma	0	0.00%	0	0.00%	0	0.00%	1	4.55%	0	0.00%	0	0.00%	1	1.00%	
NO	8	100.00%	12	85.71%	0	0.00%	0	0.00%	3	18.75%	19	100.00%	42	42.00%	
Yes	0	0.00%	1	7.14%	0	0.00%	0	0.00%	0	0.00%	0	0.00%	1	1.00%	
Unknown	0	0.00%	1	7.14%	21	100.00%	20	90.91%	13	81.25%	0	0.00%	55	55.00%	
*Any genetic disease (ID)*															
deafness + aphasia	1	12.50%	0	0.00%	0	0.00%	0	0.00%	0	0.00%	0	0.00%	1	1.00%	< 0.001**
Mental retardation	0	0.00%	0	0.00%	0	0.00%	0	0.00%	1	6.25%	0	0.00%	1	1.00%	
NO	7	87.50%	13	92.86%	0	0.00%	0	0.00%	3	18.75%	19	100.00%	42	42.00%	
Unknown	0	0.00%	1	7.14%	21	100.00%	22	100.00%	12	75.00%	0	0.00%	56	56.00%	
*HIV status*															
NO	8	100.00%	13	92.86%	0	0.00%	0	0.00%	0	0.00%	17	89.47%	38	38.00%	< 0.001**
Unknown	0	0.00%	1	7.14%	21	100.00%	22	100.00%	16	100.00%	2	10.53%	62	62.00%	

^¥^ Chi square test; * P-value < 0.05; ** P-value < 0.001

Clinical presentation with constitutional symptoms like fever, night sweats, loss of appetite, and loss of weight was observed in most patients (52%). As regards the staging of NHL, the stage of NHL in most of our patients was unknown (59%), whereas, stage IV was ranked the second most common stage (25%). In terms of the site of lymphadenopathy, generalized lymphadenopathy was the most common presentation in our NHL patients (43%). A size of > 4 cm was observed in 31% of patients, while 12% of patients had lymph node size of < 2 cm. The size of lymph nodes of most of our patients was not accessible (51%). The clinical presentation, nodal site, and size on initial presentation are summarized in (Table 2A).

**Table 2:** (A) Clinical presentation and nodal sites on initial presentation (N = 100); (B) organomegaly and extra-nodal sites on initial presentation (N = 100)

(A) Patient profile	N (%)	(B) Patient profile	N (%)
*Constitutional symptoms*		*Organomegaly (not LNs)*	
Positive (+ve)	52 (52)	Liver	23 (23)
Negative (−ve)	45 (45)	Spleen	30 (30)
Unknown	3 (3)	Pancreas	1 (1)
*Stage of NHL*	0 (0)	Cecum	1 (1)
I	1 (1)	Parotid	2 (2)
I (E)	0 (0)	Thyroid	2 (2)
I (S)	2 (2)	Kidney	1 (1)
II	4 (4)	Ovary	1 (1)
II (E)	0 (0)	Stomach	1 (1)
II (S)	3 (3)	Colon	0 (0)
III	2 (2)	Duodenum	1 (1)
III (E)	1 (1)	Brain	1 (1)
III (S)	3 (3)		
III (SE)	25 (25)	*Presentation with extranodal (primary site other than LNs)*	
IV	59 (59)	Liver	2 (2)
Unknown		Spleen	0 (0)
*Site of lymphadenopathy*		Pancreas	0 (0)
Axillary	3 (3)	Cecum	3 (3)
Supraclavicular	3 (3)	Parotid	1 (1)
Cervical	6 (6)	Thyroid	5 (5)
Mesenteric LNs	6 (6)	Kidney	2 (2)
Paravertebral	1 (1)	Ovary	1 (1)
Epigastric LNs	1 (1)	Stomach	13 (13)
Nasopharyngeal	2 (2)	Colon	2 (2)
Submandibular	3 (3)	Duodenum	1 (1)
Generalized	43 (43)	Brain	0 (0)
Retroperitoneal	1 (1)	Small bowel	3 (3)
Pre-tracheal	1 (1)	Tongue	2 (2)
Unknown	21 (21)	Cheek deep tissue	2 (2)
		Bone marrow (BM)	5 (5)
		Eyes	0 (0)
*Max. size of lymphadenopathy*		Skin	1 (1)
< 2 cm	12 (12)	Breast	2 (2)
2–4 cm	6 (6)	Bones	2 (2)
> 4 cm	31 (31)	Jejunum	1 (1)
Unknown	51 (51)	Soft tissue	1 (1)

Among patients with lymphadenopathy, extra-nodal organomegaly was observed in a total of 38 NHL patients. Of which, 23 patients presented with hepatomegaly; 30 patients with splenomegaly. Biopsy indicated that 49% of patients had primary lymphomas in the extra-nodal sites, with stomach being the most common site of primary extra-nodal NHL (13%), followed by thyroid gland, and bone marrow (5% and 5%) respectively. Extra-nodal NHL included also other regions in the body (Table 2B).

Also, mild anemia (Hb 9-12 g/dL) was detected in 42% of patients, while moderate anemia (Hb 6-8 g/dL) was detected in 14% of NHL patients. On the other hand, Leucopenia (WBC count < 4000/mm^3^) was noted in a total of 27 NHL patients, whereas, 17 patients presented with leukocytosis (WBC count > 11,000/mm^3^). Moreover, thrombocytopenia (platelet count < 150,000/mm^3^) was noted in a total of 22 NHL patients. The results of bone marrow fine needle aspiration (FNA) showed a benign lymphocytic proliferation in a total of 4 patients, while malignancy was noted in 10 cases. Noteworthy, the findings of bone marrow (FNA) was unknown in most cases (86%).

As regards typing of NHL according to IHC markers, 82 cases were CD 20-positive; 60 cases were CD 45-positive; 21 cases were CD10-positive; 19 cases were CD3-positive. Two-thirds of NHL cases were treated at our regional referral hospital while the remaining third were referred for treatment to higher centers. R-CHOP was the main chemotherapeutic regime used in 42 NHL cases. On the other hand, 7 patients were treated with other chemotherapy in conjunction with R-CHOP, while 9 patients were given chemotherapy other than R-CHOP. Upon clinical follow up, various complications were noted in 23 patients which mainly included respiratory and gastrointestinal infections (13 cases). Other complications noted included bone marrow suppression, hypothyroidism, constipation, hypertension, femur fracture, vasculitis, and Kaposi sarcoma.

### Discussion

Increased risk of NHL has been reported to be associated with various non-modifiable risk factors such as age (especially individuals of 60 years of age or more), sex, and other factor [15, 16].

According to age-standardized incidence rate (ASIR) we found that the age group of > 60 years of age had the highest incidence of NHL compared to a reported 5.5/100,000 in 2014 nationally with Aseer occupying the 5th most common region in Saudi Arabia [17]. Recently, a large investigation of various cancer registries within Saudi Arabia conducted by Rauf et al. [2] investigated NHL patients found that NHL has a peak incidence at the age group (40–60 years of age). We found that the incidence of NHL was higher among men with a M:F ratio of 1.27:1 which shows similarity to other studies [18]. Furthermore, our study revealed a steady increase in the incidence of NHL in the Aseer region: 8 cases in 2011; 14 cases in 2012; 21 cases in 2013; 22 cases in 2014. This goes in line with a recent study conducted in Saudi Arabia which incorporated the data of all NHL patients from Saudi Cancer registry [15].

The stage of NHL was unknown in most of our patients, however, we found that quarter of our population presented with stage IV NHL which was the most common among our patients. Similarly, Rauf et al. [2] reported that the majority of their NHL patients presented with advanced disease of stage IV accounting for 44% of the total population investigated. Noteworthy, the percentage of patients whose staging data were not provided in their study was only 3% while it was 59% in our case.

Gastrointestinal (GI) tract was the most commonly involved system with the stomach being the most commonly affected organ. Afterwards, Bone Marrow (BM) and Thyroid involvement was ranked the second in place. Similarly, Diab et al. [19] examined the data of 855 NHL cases and found that 41.4% of the total NHL cases were to be of primary extra nodal variety. The high incidence rate in our study as well as Rauf et al. [2] could reflect the impact of environmental factors related to the high prevalence of Helicobacter pylori infection among the Saudi population [20]. Our observation is also similar to the findings of other studies conducted in Saudi Arabia [2] and Kuwait [21, 22]. Such environmental exposures may prove daunting to determine which subtypes it can affect regionally. That being said, the rate of presentation with extra nodal NHL was much lower in studies conducted in the USA and others conducted in western countries [5–8]. Moreover, primary NHL of extra nodal variety is relatively uncommon in other parts of the world [23], while it has a surprisingly high rates in others [24]. This variation in incidence rates in different parts of the world warrants further investigation to determine the role of any attributing environmental or genetic factor.

DLBCL was the commonest type of NHL accounting for 59% of all NHL and has been found to be the most common subtype of NHL in western countries with an incidence rate of 30–40% of all cases [8, 25]. It has been found to range from an incidence rate of 20% in USA to 50.5% in Thailand [26], which is even higher in Pakistan (76.4%) [24] while in Saudi Arabia an incidence rate was reported of 6.4% in 2014 with DLBCL being the most common type similar to our finding [17].

Immunohistochemistry (IHC) remains an essential investigation for all types of lymphomas which is used for multiple purposes, which includes subtyping, prognostication, immunotherapy, differential diagnosis with other malignancies, and potentiality of targeted therapy [11, 18]. This study revealed that 82% of NHL cases were positive for CD 20 marker while 60% were positive for CD45 marker. The unavailability of some IHC markers could account for the differences in the literature [15, 18].

### Conclusions

NHL in the Aseer region has an increasing incidence over the years, particularly affecting those > 60 years of age and slightly more common among men. Patients tend to present with advanced staging with constitutional symptoms and extra nodal involvement. DLBCL is the most common subtype of NHL, while CD20 marker was significant in most cases.

## Limitations

Firstly, the small sample size of our study could limit the interpretation of our findings and not perceive them as absolute values. Furthermore, the lack of similar reports regionally makes it difficult to compare regions and their subtypes as well as the environmental exposures that may be a predisposing factor. Also, some data were not available for analysis which could potentially change the outcomes such as staging and size of NHL and other viral illnesses.

## Data Availability

The data is archived in the hospital system with the analysis and specific material and would be provided to the proper party for non-commercial use by the authors if requested.

## Abbreviations

ACH: Aseer Central Hospital
ASIR: age-standardized incidence rate
ASMR: age-standardized mortality rate
BACOP: Bleomycin, Adriamycin, Cyclophosphamide, Prednisolone and Vincristine
BM: bone marrow
CHOP: Cyclophosphamide, Adriamycin, Prednisolone, and Vincristine
CVP: Cyclophosphamide, Vincristine and Prednisolone
DLBCL: Diffuse Large B Cell Lymphoma
FNA: fine needle aspiration
GI: gastrointestinal
Hb: hemoglobin
IHC: immunohistochemistry
NHL: non-Hodgkin lymphomas
ProMACE: Procatbazine, Methotrexate, Adriamycin, Cyclophosphamide and Etoposide
U.S.A: United States
WBC: white blood cell
